# Introducing a new series in IJTLD OPEN ‘Ending the TB epidemic’

**DOI:** 10.5588/ijtldopen.24.0644

**Published:** 2025-02-01

**Authors:** H.D. Blackbourn, G.B. Migliori

**Affiliations:** ^1^ International Union Against Tuberculosis and Lung Disease, Paris, France;; ^2^ Istituti Clinici Scientifici Maugeri, Istituto di Ricovero e Cura a Carattere Scientifico, Tradate, Italy.

**Keywords:** tuberculosis, End TB, HIV, Western Pacific Region

## Abstract

The latest figures for TB are a sombre reminder that we need to accelerate progress to eradicate this disease. We are, therefore, launching a series of articles that bring the focus back onto TB elimination. We begin with an article that highlights the factors that have helped to reduce mortality due to HIV/AIDS and the lessons that might be applied to TB control. Then, the authors of a ‘Regional Perspective’ describe the current strategy to reduce the burden of TB in the Western Pacific Region. The authors critically review the progress made, and the remaining challenges, allowing them to propose additional elements to improve TB control. We anticipate that the series will highlight many commonalities, but also provide ideas for innovative solutions, and provoke discussion on how best to move forward.

Within the TB community, we often highlight positive aspects of the myriad number of initiatives for TB control. However, this is against a background of growing concern that the targets to End TB are unlikely to be met by 2030.^1–4^ Given the progress that has occurred, why – as the global figures continue to make evident – are we struggling to eliminate TB? There is a danger that we become overwhelmed by the complexity of TB diagnosis and the vast range of treatment options, and so lose clarity on the next steps. We are, therefore, launching a series of articles that focus on the bigger picture of TB elimination. Our aim is to highlight the remaining major challenges, refresh our thinking about opportunities, and provoke discussion on how best to move forward.

First, an accompanying Editorial highlights the factors that have helped to reduce mortality due to HIV/AIDS and the lessons that might be applied to TB control.^5^ Unlike for TB, HIV/AIDS has continued to decline (even during the COVID-19 pandemic).^4^ Key to this are three elements: investment in diagnostic devices led to simple tests, allowing for cost-effective mass screening; development of ART created a safe and highly effective treatment; use of prophylactics further reduced transmission. In comparison, despite significant advances, TB diagnostics remains relatively complex and difficult to deploy in the field. Similarly, TB drug regimens are complex and often require patient monitoring. These limitations are less restrictive in countries with sophisticated healthcare systems, but are a significant barrier to global TB elimination.

The second article is part of a series of Regional Perspectives that will explore these barriers, each with a candid assessment of the progress made to date and the challenges being faced.^6^ The authors describe the WHO framework,^7^ a comprehensive strategy to reduce the TB burden in the Western Pacific Region. However, its success depends on multi-sectoral partnerships, political commitment, sustained funding and health reforms. Key obstacles include undiagnosed TB cases, a lack of healthcare infrastructure, inequities in access to TB services and drug-resistant TB. The authors critically reflect on four elements: strengthening essential TB functions, building robust health system foundations, addressing social determinants of health, and enhancing governance and accountability. Their critical reflection explores these layers, and considers both the difficulty of the existing challenges, while proposing potential solutions.

We anticipate that these Regional Perspectives will identify elements that are common to each region, but also highlight many unique features and novel approaches. Once we have the full series of articles in place, we will move to a second phase and invite experts to review the best- and worst-case scenarios. The role of this expert panel will be to shine a light on the most effective approaches and propose measures that would make a meaningful difference. We hope that this will identify practical solutions to accelerate TB elimination.
